# Contributions of Traffic and Industrial Emission Reductions to the Air Quality Improvement after the Lockdown of Wuhan and Neighboring Cities Due to COVID-19

**DOI:** 10.3390/toxics9120358

**Published:** 2021-12-17

**Authors:** Xiaoxiao Feng, Xiaole Zhang, Cenlin He, Jing Wang

**Affiliations:** 1 Institute of Environmental Engineering (IfU), ETH Zurich, 8093 Zurich, Switzerland; fengx@ethz.ch (X.F.); zhanxiao@ethz.ch (X.Z.); 2 Swiss Federal Laboratories for Materials Science and Technology, 8600 Dubendorf, Switzerland; 3 Advanced Study Program and Research Applications Laboratory, National Center for Atmospheric Research (NCAR), Boulder, CO 80301, USA; cenlinhe@ucar.edu

**Keywords:** air quality, COVID-19, emission inventory, WRF-CMAQ model, sensitivity analysis, restriction policy

## Abstract

Wuhan was locked down from 23 January to 8 April 2020 to prevent the spread of the novel coronavirus disease 2019 (COVID-19). Both public and private transportation in Wuhan and its neighboring cities in Hubei Province were suspended or restricted, and the manufacturing industry was partially shut down. This study collected and investigated ground monitoring data to prove that the lockdowns of the cities had significant influences on the air quality in Wuhan. The WRF-CMAQ (Weather Research and Forecasting-Community Multiscale Air Quality) model was used to evaluate the emission reduction from transportation and industry sectors and associated air quality impact. The results indicate that the reduction in traffic emission was nearly 100% immediately after the lockdown between 23 January and 8 February and that the industrial emission tended to decrease by about 50% during the same period. The industrial emission further deceased after 9 February. Emission reduction from transportation and that from industry was not simultaneous. The results imply that the shutdown of industry contributed significantly more to the pollutant reduction than the restricted transportation.

## 1. Introduction

China has experienced severe and persistent air pollution in the past decade as a side effect of rapid economic development. The urbanization and industrialization in China not only consume a large amount of energy but also cause air pollution problems in the cities [1,2]. The concentrations of both particles (fine particulate matter less than 2.5 μm in diameter (PM_2.5_) and 10 μm in diameter (PM_10_)) and gaseous pollutants (e.g., sulfur dioxide (SO_2_), nitrogen oxide (NO_x_), and carbon monoxide (CO)) in Chinese cities have been significantly above the World Health Organization’s (WHO) recommended annual average in recent years [3]. For instance, the average levels of PM_2.5_ were five times higher than the WHO standard in 58 Chinese cities in 2013 [3,4]. 

Energy consumption, especially coal-based power that accounts for roughly 67% of the total energy, is the main source of anthropogenic emissions in China [5]. The burning of fossil fuel in the power sector pollutes the air heavily by SO_2_, NO_x_, and PM_2.5_, contributing roughly 33%, 33%, and 6% of the country’s total emissions of SO_2_, NO_x_, and PM_2.5_, respectively [6,7]. As a result of rapid industrialization, the industry sector holds a large share in the energy consumption structure and is one of the main contributors to the air pollution in China [8]. Meanwhile, vehicle density has significantly increased and vehicle exhaust has also aggravated Chinese air pollution [5]. Air pollution poses a major threat to health and climate [9,10,11]. To control the air pollution, Chinese authorities have already spent tremendous efforts and issued policies to limit the emissions from the power, industry, and transportation sectors [12], but it is hard to compensate for the effects of the economic growth and increasing usage of fossil fuel. 

Wuhan is one of the metropolises and the most populous city in Central China as well as the capital of Hubei Province. It is located at the junction of the Yangtze River and Hanjiang River, functioning as an important transportation hub and industrial center. The industrialization and commercial trade inside and outside the city brought Wuhan a fast growth in GDP, which was 7.8% in 2019 and 1.7% higher than the national average [13]. However, the rapid economic development also causes air quality problems around Wuhan. Research showed the annual PM_2.5_ concentrations were 106.5 to 114.9 μg m^−3^, with sulfate, nitrate, ammonium, and organic matter as dominant components in 2013. The peak value of air pollution occurred in December as a result of increased local emissions, low temperature, low wind speed, and high atmospheric pressure. The emissions from industrial activities accounted for 34% of secondary particulate matter, 57% of primary dust, and 45% of total SO_2_ emissions in Wuhan [14].

The novel coronavirus disease 2019 (COVID-19) is an infectious disease caused by SARS-CoV-2, breaking out in December 2019 [15,16], which has spread globally. To prevent the spread of virus, countries around the worlds adopted different approaches. Towns and cities were locked down and large gatherings were banned. Restrictions on traffic were imposed in a few countries, such as China, India, and Iran [17,18,19,20,21]. Many non-essential human activities were also limited. The air quality was improved during the worldwide lockdown as a result of the reduced polluting source, related to less human activity. Reduced aerosol pollution was reported in India, Italy, the UK, etc. [17,18,19,20,21,22]. The gaseous pollution, such as NO_x_, was also reduced worldwide [23]. To control the spread of the coronavirus and quarantine the identified epicenter of the outbreak, Wuhan, the Chinese authorities announced the lockdown of Wuhan city on 23 January 2020. All public transportation was suspended to cut off the impact of the disease outside the city, including buses, railways, highways, flights, and ferry services. The Wuhan airport, railway station, and metro station were closed. The citizens of Wuhan were not allowed to leave the city without permission from the authorities [24]. The lockdown was further applied to 16 neighboring cities in Hubei Province, such as Huanggang, Xiaogan, and Suizhou. Traffic restrictions were also applied in the quarantined cities. The manufacturing industry was impeded by the lockdown. Wuhan has large-scale industrial clusters in the electronics, automotive, and pharmaceutical fields. The lockdown exerted a significant impact on the production process and product delivery. 

The lockdown of Wuhan and surrounding cities provided an unintended experimental condition under which to investigate the influences of emissions from different sectors, e.g., traffic and industry, by completely or partially removed emissions. A few studies showed that the air pollution level declined during the lockdown period [25,26,27,28,29,30]. The air pollution reduction can be visualized from measurements by the China National Environmental Monitoring Network [31]. However, the measurements only provide total concentrations of pollutants, not detailed information that can be used to explore the reason for air pollution reduction, such as the sector-specific contribution to the air pollution.

Air quality modeling is a powerful tool for reproducing and predicting air pollution at diverse scales. There are multiple applications of numerical models, such as analysis of physical processes [32,33,34], pollution forecasts [35,36,37,38], sensitivity analyses [39,40,41], and inverse modeling [42,43,44,45,46,47,48,49]. While the observations can only provide limited information on air pollution, the air quality model can be used as an analytical tool for supplementing the details. For instance, the model-based source apportionment method can be used to analyze the contributions of different sectors to air pollution [50]. Sensitivity analysis can be conducted to estimate the change in pollutant concentrations associated with the change in emissions. 

This study analyzed the influence of the lockdown of Wuhan city on the local air quality. The contributions of transportation and industry sectors were calculated based on air quality model analyses. The change in air pollution in Wuhan was discussed by comparing the simulation results and the measurement data from ground stations.

## 2. Data and Methods

### 2.1. Air Quality Monitoring Data

**Figure 1:**
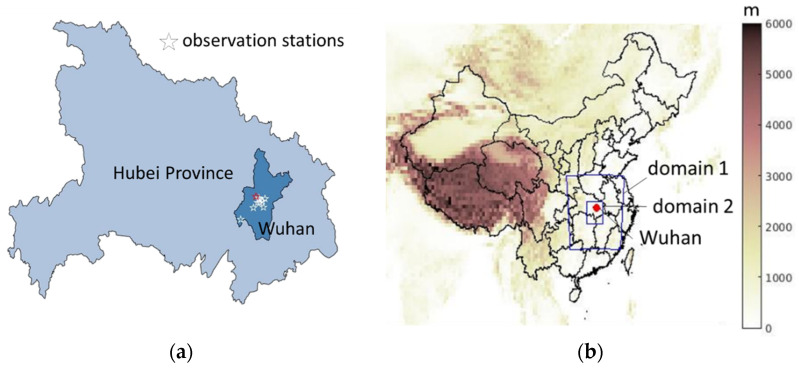
(**a**) Locations of 10 observation stations. The stars refer to 10 observations stations in Wuhan, including the representative Tianhe station (red star). (**b**) Model domains. The blue profile refers to terrain heights of the two nested model domains with horizontal resolutions of 10 km × 10 km (domain 1), 5 km × 5 km (domain 2). The red point is the location of Wuhan.

The measurement data were collected from the stations of the China National Environmental Monitoring Network. The Monitoring Network offers an air quality index (AQI) as well as hourly and daily average concentrations of PM_2.5_, PM_10_, SO_2_, NO_2_, and CO at different locations in China from 2014 to the present. Figure 1a shows the geographical locations of the 10 monitoring stations in Wuhan, with the distance of about 10 km between each other. Since the monitoring stations are close, the observational data did not vary abruptly from each other. The standard deviations of pollutant concentrations among the 10 stations were relatively small, and the temporal trends of pollutant concentrations were similar. For instance, the standard deviations were around 10.3 µg/m^3^, 8.0 µg/m^3^, 1.4 µg/m^3^, 8.4 µg/m^3^, and 0.2 mg/m^3^ for PM_2.5_, PM_10_, SO_2_, NO_2_, and CO during 10 January and 15 February. Data from the Tianhe observation station were close to the mean concentration recorded by the 10 stations and therefore were representative. The type of monitoring station was the urban traffic station. Table 1 shows the measurement methods and the pollution limits according to China’s ambient air quality standards GB 3095–2012. Satellite data can also be used to compare the air quality before and after the lockdown. Additional satellite data are given in the Supplementary Materials.

The impact of lockdown was investigated using the observations. Meanwhile, model simulations (Section 2.3) were also validated by comparing with the observations, and more detailed information was analyzed using the simulations. 

### 2.2. Significance Test

Before analyzing the impact from different sectors on air pollution, it is necessary to perform the significance tests and examine the changes of pollutant concentrations (PM_2.5_, PM_10_, SO_2_, NO_2_, and CO) before and after the lockdown based on the ground measurements.

The Mann–Kendall test is a non-parametric test for identifying trends in time series data. It is widely employed in analyses of environmental, meteorological, and hydrological data. The test compares the relative magnitudes of sample data rather than the data values themselves [51]. The data applied in the Mann–Kendall test can be any particular distribution and do not need to be normally distributed. Moreover, the test is tolerant of non-detected data by assigning them a common value that is smaller than the smallest measured value in the data set. The temporal data sequence should consist of one data point for a certain time period. 

The Mann–Kendall statistic, *S*, is normally distributed. The calculation of *S* is shown in Equation (1). The initial value of *S* is set to be 0. Each data point in the temporal sequence is compared with its former data point. If a datum’s value is higher than the value of the former data point, *S* is incremented by 1. On the contrary, *S* is decremented by 1. *S* with a high positive/negative value indicates an increasing/decreasing trend of the temporal sequence. *U*_k_ is the standard normal distribution converted from *S*, while *U*_b_ is the standard normal distribution converted from the reverse sequence of *S*. The calculation of *U*_k_ and *U*_b_ is shown in Equation (2). If the intersection point of line *U*_k_ and *U*_b_ exists, a significant change is considered to occur.

(1) $$\begin{array}{l} S=\sum_{k=1}^{n-1}\sum_{j=k+1}^{n}sign(x_{j}-x_{k})\\ sign(x_{j}-x_{k})=\{\begin{array}{c} 1\;\mathrm{if}\;x_{j}-x_{k}>0\\ 0\;\mathrm{if}\;x_{j}-x_{k}=0\\ -1\;\mathrm{if}\;x_{j}-x_{k}<0 \end{array} \end{array}$$

(2) $$U=\{\begin{array}{cc} \frac{S-1}{{\left[VAR(S)\right]}^{1/2}} & \;\mathrm{if}\;S>0\\ 0 & \;\mathrm{if}\;S=0\\ \frac{S+1}{{\left[VAR(S)\right]}^{1/2}} & \;\mathrm{if}\;S<0 \end{array}$$

### 2.3. Air Quality Model

The combined Weather Research and Forecasting Model (WRF) version 3.8 and Community Multiscale Air Quality Modeling System (CMAQ) version 5.2 were used in this research. Driven by the meteorological field generated by the WRF model, the CMAQ system calculated the pollutants’ formation, transport, evolution, and removal. 

In the WRF-CMAQ model, two nested domains were set up using one-way nesting in the Lambert Conic Conformal projection with horizontal resolutions of 10 km × 10 km and 5 km × 5 km, respectively, shown in Figure 1b. The larger domain covered the Hubei Province and some parts of the neighboring provinces. The smaller domain mainly covered Wuhan City. The simulation utilized 30 terrain-following σ-levels up to 10 hPa (i.e., ~20 km a.s.l.). The land use information was obtained from the MODIS (Moderate Resolution Imaging Spectroradiometer) IGBP (International Geosphere–Biosphere Programme) 21-category data [52].

To configure the WRF model, the WRF Single-Moment (WSM) 3-class simple ice microphysics scheme, Rapid Radiative Transfer Model (RRTM) scheme, Dudhia scheme, Kain–Fritsch (new Eta) scheme, Yonsei University (YSU) planetary boundary layer (PBL) scheme, and Noah land-surface model were adopted. WRF was initialized by the meteorological field from the final global tropospheric analyses by National Centers for Environmental Prediction (NCEP) Global Forecast System (GFS). The NCEP GFS data also provided the boundary conditions for the WRF model. For the CMAQ model, the boundary conditions of domain 1 were given by the default values embedded in CMAQ, while the boundary conditions of domain 2 were derived from the simulation results of domain 1. The initial conditions were also from the default initial values listed in CMAQ. To reduce the influences of the initial conditions, the first 3 days were regarded as the ‘spin-up’ period of the simulation [53]. The chemical mechanisms used in the model were CB05e51 (Carbon Bond 2005 e51) and aerosol6 [54]. The simulation period was from 10 January 2020 to 15 February 2020, in total 37 days, which covered 14 days before the city lockdown and 23 days after the lockdown.

### 2.4. Emission Inventory for Wuhan

Emission inventory is the indispensable data for air quality models. It contains the information on emission rates of different species, such as PM_2.5_, PM_10_, NO_x_, SO_2_, etc. This study employed the Multi-resolution Emission Inventory for China version 1.3 (MEICv1.3) as the emission inventory. MEIC is a static anthropogenic emission inventory with the resolution of 0.25° × 0.25°, including the emission information from five different sectors in China, i.e., power sector, industry sector, residential sector, transportation sector, and agriculture sector. The time coverage of MEIC ranges from 2008 to 2016. In this study, the most recent emission inventory of 2016 was adopted. Since the emission inventory is static and might not be capable of describing the scenarios in 2020 accurately, MEIC was updated in our study to approach the actual emission. The detailed method is given in the Supplementary Materials.

Sparse Matrix Operator Kerner Emissions Modelling System (SMOKE) version 4.5 was used as the preprocessor to prepare the baseline MEIC emission inventory [55]. The emission inventory was re-gridded to match the spatial and temporal configurations of CMAQ. The pollutants from the emission inventory were classified into more specific species to fit the CMAQ chemical mechanism (cb05e51_aero6). 

The lockdown of Wuhan and the nearby cities were implemented after 23 January, and the intra-city and inter-city transportation was suspended. The quarantined area covered the whole province except for Shennongjia Forest District.

In the study, four scenarios were designed to investigate the impact of the transportation sector and industry sector on air pollution during the lockdown. The pollutant concentrations were simulated under the following scenarios with the updated MEIC emission inventory.

(A) Business as usual: it was assumed that the cities continued normal activities and there was no lockdown in Hubei Province. Emissions in the period were from the industry, transportation, residential, power, and agriculture sectors in the updated MEIC emission inventory.

(B) Without traffic emission: It was assumed that there was no emission from transportation in the simulation domains 1 and 2 after the lockdown of Wuhan and surrounding cities. The emissions from the industry, residential, power, and agriculture sectors were kept the same as in scenario (A).

(C) Without traffic and half industrial emissions: after lockdown of Wuhan, it was assumed that there was no emission from transportation, and the industrial emission was reduced by 50% in the simulation domains 1 and 2. Emissions from residential, power, and agriculture sectors were kept the same as in scenario (A).

(D) Without traffic and industrial emissions: it was assumed that there was no emission from transportation or industries in the simulation domains 1 and 2. Emissions from residential, power, and agriculture sectors were kept the same as in scenario (A).

## 3. Results

### 3.1. Meteorological Data

**Figure 2:**
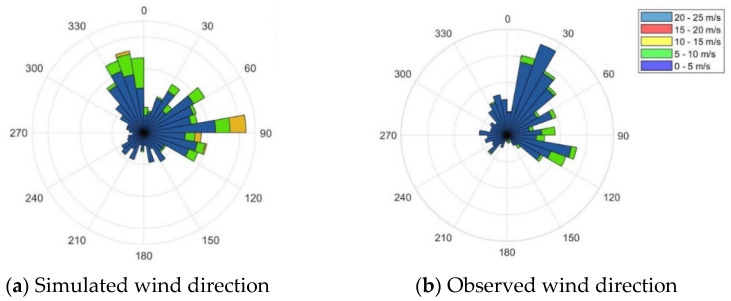

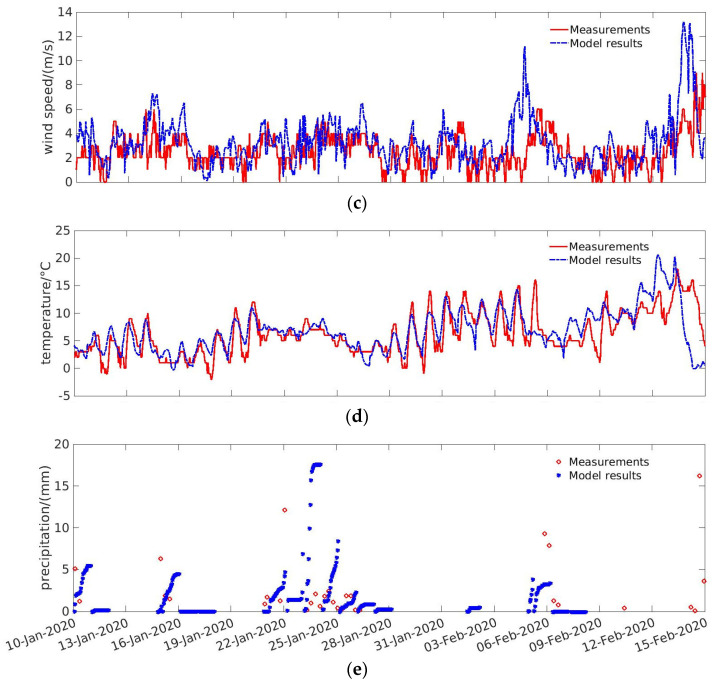
Meteorological parameters. The four figures show comparisons of measured and modelled (**a,b**) wind direction, (**c**) wind speed, (**d**) air temperature, and (**e**) precipitation at Tianhe Station between 10 January and 15 February.

The meteorological data produced by WRF were evaluated first since they were the driving force of pollutant transport. Simulation results were compared with the observations at Tianhe Station at Wuhan Airport, shown in Figure 2. Before the lockdown, the ambient temperatures were between 0 °C and 10 °C. The temperatures rose approximately by 5 °C afterwards, shown in Figure 2d. The northeast wind was dominant in January and February, and the measured wind speed was moderate, below 6 m/s, shown in Figure 2a–c. The wind speed was low between 9 February and 14 February, but the pollution was not severe, indicating the emission might be lower than before. Influenced by the cold front moving from the north, several rapid light rains occurred, shown in Figure 2e. The temperature decreased along with the precipitation in a short time. The measured meteorological variables did not change strongly before and after the city lockdown. 

The modeled wind velocity agreed reasonably well with the measurements, but the simulation overestimated the wind speed on 4 February and 13 February. The calculated wind direction basically agreed with the observed wind direction. The simulated and observed prevailing wind came from the north in general. The modeled temperature captured the characteristics of the measurements.

### 3.2. Air Quality Measurements

**Figure 3:**
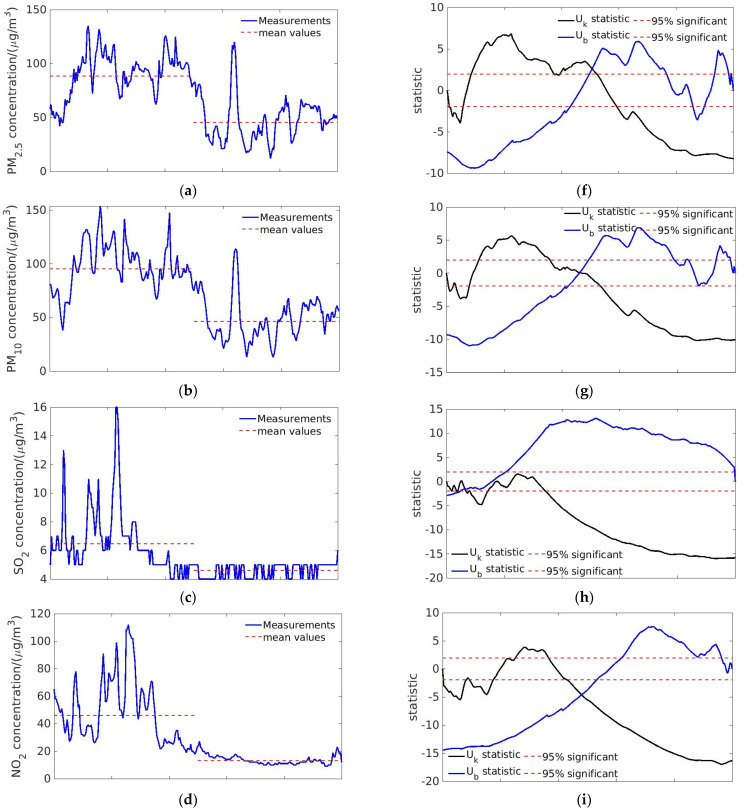

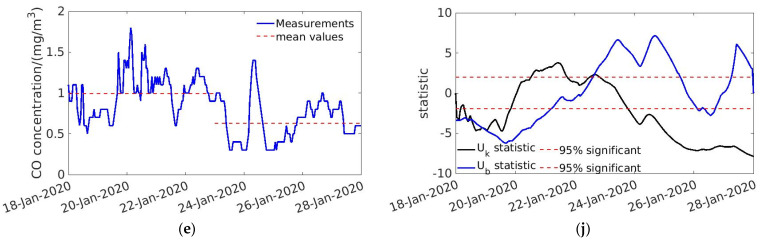
Significance test. Figures show the measured concentrations (blue lines) of (**a**) PM_2.5_, (**b**) PM_10_, (**c**) SO_2_, (**d**) NO_2_, (**e**) CO at Tianhe Station between 18 and 28 January. The red dash lines refer to the mean concentration before and after the lockdown. (**f**−**j**) shows the significance tests of concentration sequence corresponding to the pollutants.

Significance tests were conducted to analyze the changes in pollutant concentrations as a result of the lockdown based on the Mann–Kendall test and ground measurements. The temporal sequences of PM_2.5_, PM_10_, SO_2_, NO_2_, and CO were measured at Tianhe Station, from 18 to 28 January 2020, shown in Figure 3a–e. The significance tests are shown in Figure 3f–j. The mean value and the amplitude of the temporal series of NO_2_ concentration varied significantly before and after the lockdown of Wuhan and surrounding cities. It should be noted that meteorological conditions should also be taken into consideration. The meteorological condition did not change much during the considered period and was not the main reason for the change in the NO_2_ concentration. In Section 3.4 and Section 3.5, we further show that the simulated concentrations by WRF-CMAQ system considering the effect of the weather conditions agreed well with the measurements before the lockdown but became significantly higher than the observations (especially after 9 February) if the emission remained, which indicated that meteorological condition was not the primary cause for the alleviation of pollution.

**Figure 4:**
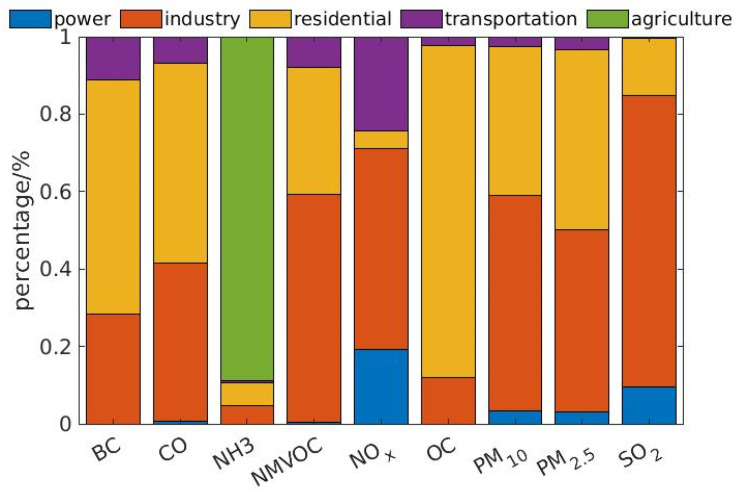
Emission inventory. The histogram shows the percentage of emission rate for the air pollutants in power, industry, residential, transportation, and agriculture sectors in Hubei Province.

The Mann–Kendall test for NO_2_ denoted a changing point on 23 January, shown in Figure 3i. The value of *U*_k_ was negative and out of the 95% confidence interval, demonstrating the statistically significant decrease in NO_2_ concentration after locking down the cities. Since the transportation sector and industry sector accounted for nearly 80% of the primary NO_x_ emission (Figure 4), the sensitivity analysis for transportation and industry sectors was conducted as described in Section 3.5 and Section 3.6. Similarly, the concentrations of PM_2.5_, PM_10_, and CO decreased after 23 January, shown in Figure 3a,b,e, corresponding to the changing point in the significance tests, shown in Figure 2f,g,j. In Figure 3c, the temporal series of SO_2_ concentration had several peaks before the lockdown, while the sequence flattened afterwards. However, the significance test of SO_2_ did not show a statistically significant change around 23 January, shown in Figure 3h. 

### 3.3. Emission Analysis

To have an overview of the annual emission in different sectors in Hubei Province, the data from the MEIC emission inventory were collected and summarized in Figure 4, which shows that industry and residential sectors contributed most to the total emissions in Hubei Province. Industry was the main source of SO_2_ (75.2%), NO_x_ (51.8%), CO (41.0%), VOC (58.8%), and the average of PM_2.5_ and PM_10_ (51.4%). The transportation sector accounted for 24.2% of NO_x_ emissions, while accounting for equal or less than 11% of other species emissions. Most NH_3_ was emitted from agriculture activities. 

### 3.4. Model Results and Validation

Air quality models, as source-oriented methods, are currently practical and low-cost ways to evaluate the effects of emissions. However, the results of air quality models may have notable discrepancies from the ambient measurements. The difficulties of acquiring accurate simulation results are due to the inaccuracy of the model inputs, including emission inventories, meteorological conditions, and the incomplete knowledge about the physical and chemical processes. The emission inventory is the most direct information to estimate the pollution, and it also has large uncertainty. MEIC v1.3 is the emission inventory based on the investigation from 2016. Therefore, discrepancies were anticipated from the actual conditions in 2020.

Before locking down cities in Hubei Province, it was assumed that all sectors had normal activities. The MEIC emission inventory for all sectors was first preprocessed with SMOKE and applied in the CMAQ model without any modification. The simulation results from CMAQ from 10 January to 23 January were compared with the ground observations to validate the air quality model, named the baseline MEIC case. Afterwards the MEIC emission data were updated to reduce the discrepancies between the simulations and observations. The update method and detailed results are given in the Supplementary Materials.

Before updating MEIC, the concentrations of modeled PM_2.5_ and PM_10_ were approximately half of the observations, while the temporal trends of the daily variations were basically consistent with the observations. The CMAQ model overestimated the concentration of SO_2_ by 250%, and the daily variation was larger than the observations. CMAQ also underestimated the concentrations of NO_2_ and CO by approximately 60% and failed to capture several extremely high peaks. The discrepancies were mainly caused by the outdated emission inventory. 

The CMAQ simulation with the updated emissions showed a much better agreement with the observations than the baseline case. After adjusting the emissions, the concentrations of PM_2.5_ and PM_10_ were around 2.5 times higher than the baseline case and much more consistent with measurements. SO_2_ was relatively stable after it was emitted into the atmosphere, so the concentration of SO_2_ was reduced as the same ratio of the reduction in the emission rate. The NO_2_ and CO concentrations increased significantly by a factor of 4 and 3.5, respectively.

The simulation results were improved and had a comparable average as the observations after emission update. Therefore, the modified MEIC emission inventory was used to analyze the impact from different sectors.

### 3.5. Impact of Transportation Sector on Air Pollution

In recent years, the concern about exhaust emissions from motor vehicles has been increasing. Transportation is believed to be a major contributor to air pollution, especially for NO_x_ concentration [39]. The diesel engines have high emissions of NO_x_ and particulate matter. Since the transportation both in Wuhan City and Hubei Province was suspended right after the lockdown, it was possible to evaluate the transportation emission trends and associated air quality impact. 

**Figure 5:**
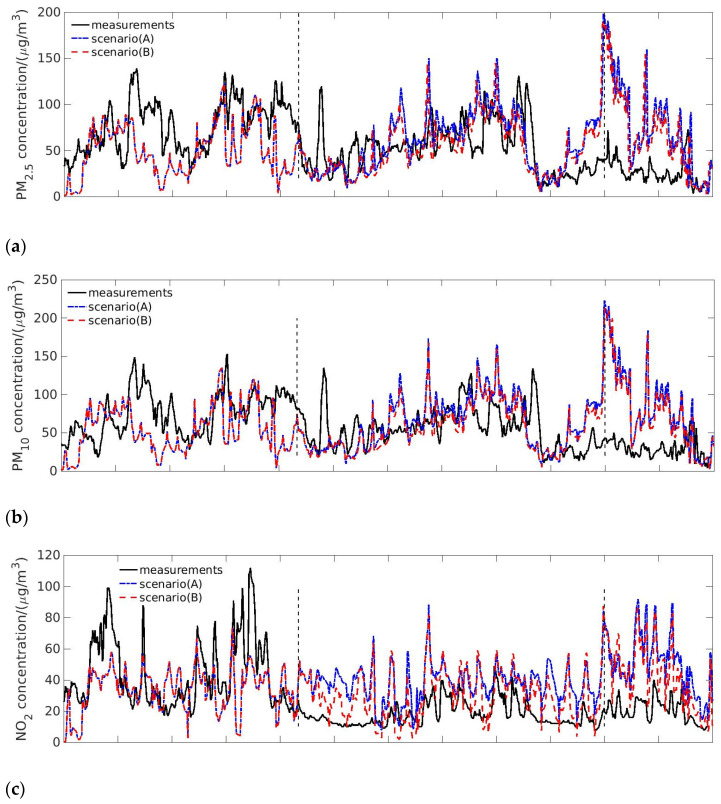

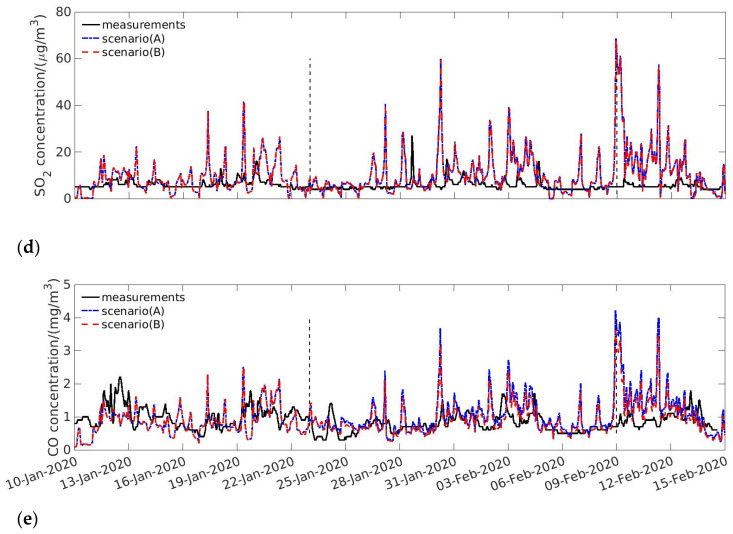
Pollutant concentrations influenced by the transportation emission. Each panel shows the comparison of observations and simulation results of scenario (**A**) and (**B**) for (**a**) PM_2.5_, (**b**) PM_10_, (**c**) NO_2_, (**d**) SO_2_, and (**e**) CO.

Scenario (A) ‘Business as usual’ and Scenario (B) ‘Without traffic emission’ after lockdown were designed to evaluate the influence of the transportation sector on air pollution. We compared the results of scenario (A) with scenario (B), and the simulation results are given in Figure 5.

Figure 5a,b show the measurements from Tianhe monitoring station and simulation results of Scenario (A) and (B) regarding PM_2.5_ and PM_10_ concentrations. Before 23 January, the meteorological and emission conditions for scenario (A) and (B) were the same. Therefore, the simulation results showed no differences. After 23 January, the concentration differences between Scenario (A) and (B) for PM_2.5_ and PM_10_ were not significant, even though the transportation emission was completely removed in Scenario (B). The reduction ratios of PM_2.5_ and PM_10_ were respectively around 9.7% and 9.0%. However, the observation showed a decrease by 40% of PM concentration after the lockdown and a further decrease by 70% after 9 February. Compared with the observations, the simulation results of both Scenario (A) and (B) were slightly higher after 23 January, but they were much higher after 9 February. The results indicated that the suspension of transportation contributed to only a portion of the PM_2.5_ and PM_10_ reduction from 23 January to 8 February; the fraction of this contribution to the PM reduction after 9 February was even smaller.

Figure 5c shows the temporal trends of NO_2_ concentrations from measurements in the monitoring station and the simulation results, where differences between scenario (A) and (B) were observed following the lockdown. After removing the transportation emission, the NO_2_ concentration decreased by 18.4% on average. However, the calculated NO_2_ concentrations were still higher than the observations. Therefore, the transportation emission was insufficient to completely explain the reduction in NO_x_ pollution. 

Figure 5d shows the observed SO_2_ concentration and the simulation results in scenario (A) and (B). SO_2_ emission from transportation sector was quite limited, less than 2%. As a result, the transportation sector had limited influence on the SO_2_ concentration, and there was no significant difference between the simulation results of scenario (A) and (B). Meanwhile, the SO_2_ concentration was higher than observations after the lockdown, which meant the emission from other sectors had been overestimated. The comparison between simulation results and observations of CO were parallel to that of particulate matter, shown in Figure 5e. Without the transportation emission, the average concentration of CO decreased by 13.4%, while the contribution of CO emission from the transportation sector was around 10%. The simulated concentration of CO was comparable with the observations before 9 February, but higher than the observations afterwards.

In summary, transportation emission had an influence on air quality, especially on NO_x_. From 23 January to 8 February, the pollution reduction after the lockdown was partially caused by transportation restriction. However, After 9 February, the improvement in air quality was mainly caused by the emission reduction from other sectors.

### 3.6. Impact of the Industry Sector on Air Pollution

The GDP of Wuhan and Hubei Province fell by 40.5% and 39.2% in the first quarter year-on-year growth [56]. The lockdown of the city and the province had a substantial impact on industry. Factories were shut down or operated in reduced capacity. As a result, the emission reduction from industries should also be considered. In contrast, power systems usually supply a large amount of uninterrupted power, and the emissions from the power sector hardly change significantly. Meanwhile, the total amount of emission from power sector was significant less than the industry sector (Figure 4). Furthermore, the power sector contributed mainly to the emission of SO_2_ and NO_x_, but SO_2_ did not show a significant change after the quarantine. Therefore, we did not consider the emission change from the power sector. The emissions from residential and agriculture sectors were assumed to stay constant because the basic life of residents continued during the quarantine. 

Since the lockdown exerted a significant impact on the large-scale industrial clusters and since industrial emissions contributed a large fraction to all pollutants, except for NH_3_, it was necessary to analyze the sensitivity of the industrial emissions and quantify its influence on air quality. Two scenarios were investigated in the section, which were Scenario (C) ‘Without traffic and half industrial emissions’ and Scenario (D) ‘Without traffic and industrial emissions’.

**Figure 6:**
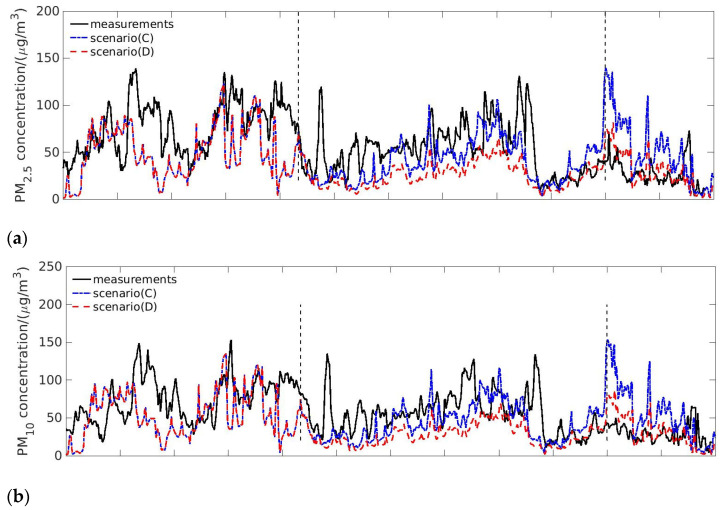

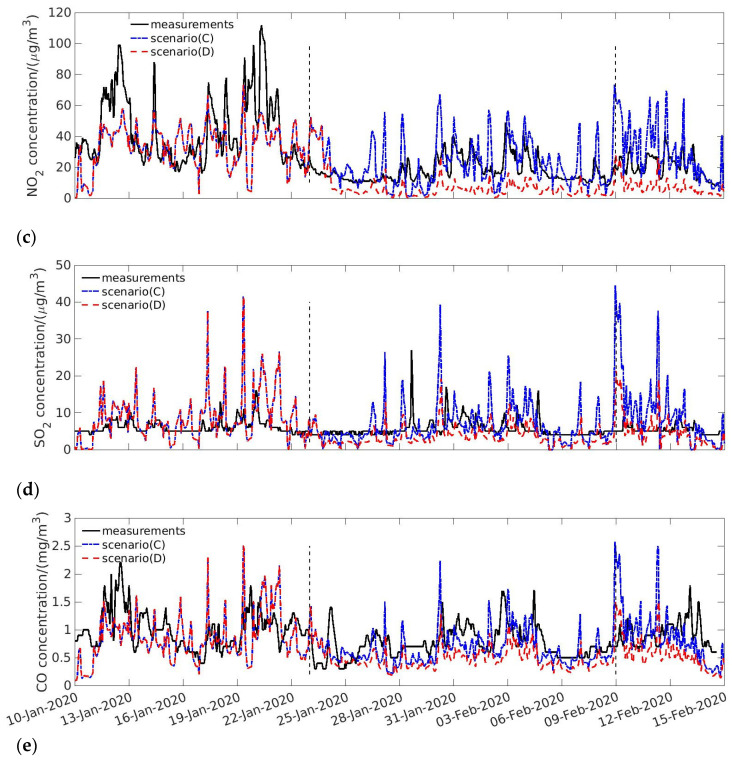
Pollutant concentration influenced by the transportation and industry emissions. Each panel shows comparison of observations and simulation results of scenarios (C) and (D) for (**a**) PM_2.5_, (**b**) PM_10_, (**c**) NO_2_, (**d**) SO_2_, and (**e**) CO.

Figure 6a,b shows the comparisons between the observations and the simulation results of PM_2.5_ and PM_10_ concentrations in scenarios (C) and (D). After removing 50% industry emission and all the transportation emission, the particulate concentration was significantly lower than the case only removing the transportation emission (scenario (B)), with a reduction of 25.0% for PM_2.5_ and 25.6% for PM_10_ compared with Scenario (B). When the total industry emission was removed, the concentrations of PM_2.5_ and PM_10_ decreased further, with a reduction of 53.8% for PM_2.5_ and 54.8% for PM_10_ compared with scenario (B). Scenario (C) and (D) underestimated the observed PM_2.5_ and PM_10_ concentrations before 9 February. Scenario (C) was closer to the observations from 23 January to 8 February, while scenario (D) agreed better with the observations afterwards. Both cases captured some characteristics of temporal evolution of PM_2.5_ and PM_10_ concentrations after the lockdown.

The simulation results for NO_2_ under scenarios (C) and (D) are shown in Figure 6c and are compared with observations. The concentration of NO_2_ decreased by 30.0% after removing 50% of industry emission and total transportation emission, compared with the case in which only the transportation emission was subtracted (scenario (B)). When all of the industry emissions were removed, the concentration of NO_2_ further decreased by 72.1% compared with scenario (C) and by 80.4% compared with scenario (B). In general, the scenario with 50% reduction in industry emission and total transportation emission removal agreed better with the observations, but there were still peaks that did not occur in the measurement. The scenario removing all the industry and transportation emissions (scenario (D)) underestimated the observed NO_2_ concentration after the lockdown continuously.

Reducing the industry emission had an impact on SO_2_ concentrations. The simulation results of SO_2_ in scenario (C) and (D) and the observations are shown in Figure 6d. The industry emission of SO_2_ accounted for 59% of total SO_2_ emission; thus, the SO_2_ concentration decreased sharply after removing the industry emission. The SO_2_ concentration decreased by 32.9% and 65.2% after removing half and all of the industry emission, respectively, compared with the scenario only removing transportation emission (Scenario (B)). Removing half of the industry emission after the lockdown led to higher SO_2_ concentration than observations, especially after 9 February. When the total industry emission was removed, the SO_2_ concentration became underestimated. Figure 6e shows the simulation results of CO under scenarios (C) and (D). Similarly, the CO concentration decreased with partially and totally removed industry emissions, and scenario (C) showed better agreement with the observations. 

## 4. Discussion

We collected the observed data in January 2018 and 2019 in Wuhan. Compared with the average of pollutant concentrations in 2018 and 2019, PM_2.5_, PM_10_, SO_2_, NO_2_, and CO decreased significantly by 40.3%, 38.1%, 71.0%, 28.6%, and 17.3%, respectively, in the same period (from 23 January to 15 February) during the lockdown in 2020. The comparison between data from previous years and 2020 showed a significant decline in all the pollutants during the lockdown. Meanwhile, researchers investigated the trend of pollutant concentrations from 2016 to 2020 around the world [17,18,57,58]. Most pollutants decreased in all the continents. For instance, the average monthly ground-level PM_2.5_ concentration decreased by 10.6% and 26.8% globally in January and February relative to the 5-year average for the same month [57,58]. Meanwhile, global NO_2_ concentration decreased by 13.5% and 31.8% in January and February [57,58]. The decline in PM_2.5_, PM_10_, and NO_2_ in Wuhan during the lockdown was higher than the world average.

**Figure 7:**
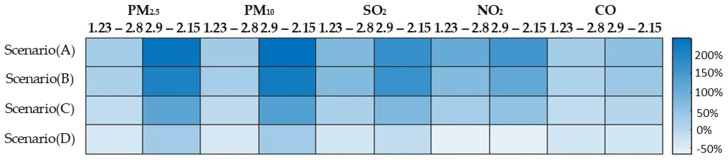
Comparison of Scenarios (A), (B), (C), and (D) with observations. The color refers to (observation − simulation)/observation.

The differences between the four scenarios and observations are summarized in Figure 7. The relative difference was defined as the ratio of the difference between the simulation and observation to the observation values. For PM_2.5_ and PM_10_, scenario (C) agreed well between 23 January and 8 February, and scenario (D) captured the observed characteristics afterwards. For NO_2_ and SO_2_, scenario (C) overestimated the concentrations, while scenario (D) underestimated the concentrations after the lockdown. Scenario (B) and (C) described the CO concentration variation reasonably. Overall, scenario (C) agreed best from 23 January to 8 February. The emission subtraction from transportation and reduction from industry was the major reason for the air quality enhancement. After 9 February, scenario (D) captured the main characteristics of the air quality. The industrial emission reduction contributed much more to the air quality improvement than transportation. The four scenarios from 23 January to 15 February were compared statistically. The Pearson correlation and the root-mean-square deviation (RMSD) were calculated for each pollutant in the four scenarios. From 23 January to 8 February, scenario (C) showed the best agreement with the observations, except for SO_2_ during the lockdown. After 9 February, scenario (D) agreed well with the observations. The point-to-point comparison figures are shown in the Supplementary Materials.

**Figure 8:**
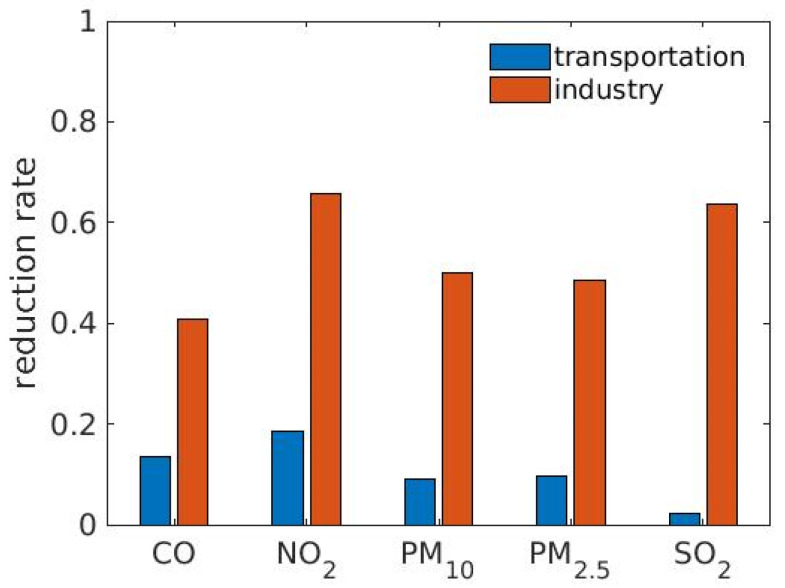
The reduction rate of pollutant concentrations due to the subtraction of transportation and industry emissions. Blue columns refer to the reduced pollution by removing all the transportation emission. Red columns refer to the reduced pollution by removing all the industry emission.

The reduction rate of pollutant concentrations caused by transportation and industry is shown in Figure 8. Removing the transportation emission was estimated to cause the decline in PM_2.5_, PM_10_, SO_2_, NO_2_, and CO concentrations by 9.7%, 9.0%, 2.2%,18.4%, and 13.4%, respectively. The total industrial emission reduction would be responsible for 48.5%, 50.0%, 63.8%, 65.6%, and 40.8% of pollution reductions in PM_2.5_, PM_10_, SO_2_, NO_2_, and CO, respectively, after the lockdown of Wuhan and surrounding cities.

Our approach of choosing the transportation and industry sectors and conducting emission reductions had limitations. Though the emission reduction corresponded to the transportation restriction and the reduction in industrial activities, which was reported by others as well [59], none of the four cases in our study reflected accurately the real emission reductions during the lockdown in Wuhan.

## 5. Conclusions

We used both measurements and model simulations to investigate the possible reason for the air quality improvement as a result of the lockdown of Wuhan and neighboring cities in Hubei Province after the outbreak of COVID-19. The emissions from transportation and industry sectors were taken into consideration since the transportation in Hubei Province and some industrial production activities were suspended after 23 January to prevent the spread of virus. The CMAQ results of four scenarios were compared with the observations.

Analysis of the observations confirmed that the temporal change in air pollutants PM_2.5_, PM_10_, NO_2_, and CO right before and after the lockdown was statistically significant. 

Scenario (A) assumed normal emissions from transportation, industry, power, agriculture, and residential sectors; the simulation results showed higher pollutant concentrations compared with the observations, which implied a reduction in emission in the real condition. Since transportation was suspended, the total emissions from the transportation sector were removed from scenario (B). The concentrations for the pollutants decreased, especially for NO_2_, and the simulation results were slightly higher than observations from 23 January to 8 February, with average differences of 13.7%, 18.8%, and 10.6% for pollutants PM_2.5_, PM_10_, and CO, respectively. Meanwhile, scenario (B) overestimated the concentration of SO_2_ and NO_2_ by more than 50%. After 9 February, scenario (B) overestimated all the pollutant concentrations. Therefore, the reduction in transportation emission was the one of the factors that improved air quality after the lockdown of Wuhan and surrounding cities. 

In scenario (C) and (D), 50% and 100% of industry emission was subtracted from the total emission, respectively, to consider different degrees of industry shutdown. Scenario (C) showed the best agreement with the observations for PM_2.5_, PM_10_, and SO_2_ before 9 February with a difference of 13.6%, 10.1%, 15.1%; for NO_2_ with a difference of 18.0% and 47.5% before and after 9 February; and for CO with a difference of 1.3% after 9 February. Meanwhile, Scenario (D) had the best agreement after 9 February for PM_2.5_, PM_10_, and SO_2_ with a difference ratio of 28.0%, 31.1%, and 12.6%, respectively. The simulation results indicate that emissions from the industry sector reduced during the quarantine period, especially after 9 February, and imply that the reduction in transportation and industry emission was not at the same pace.

In summary, this study evaluated the emission reduction from transportation and industry sectors and the associated air quality impact. The results indicate that the reduction in traffic emission was nearly 100% immediately after the lockdown between 23 January and 8 February and that the industrial emissions tended to decrease by about 50% during the same period. The industrial emissions further decreased after 9 February. The results also imply that the shutdown of industry contributed significantly more to the pollutant reduction than the restricted transportation.

## Figures and Tables

**Table 1:** Measurement methods and the upper limit concentrations of pollutants.

Pollutants	Measurement Method	Upper Limit Concentration(μg/m^3^) 24-h Average	
		1st Class	2nd Class
PM_2.5_	tapered element oscillating microbalance / Beta-ray method	35	75
PM_10_	tapered element oscillating microbalance / Beta-ray method	50	150
SO_2_	UV fluorescence analyzer / differential optical absorption spectroscopy	50	150
NO_2_	chemiluminescence analyzer / differential optical absorption spectroscopy	80	80
CO	Non-dispersive infrared absorption method	4000	4000

## Data Availability

The datasets used and/or analyzed during the current study are available from the corresponding author on reasonable request.

## Supplementary Materials

The following are available online at https://www.mdpi.com/article/10.3390/toxics9120358/s1, Figure S1: Satellite images. The red profiles refer to tropospheric columns of NO_2_ observed by OMI over Hubei Province on 21, 26, and 28 January, Figure S2: Model validation. Figures above compared the observation (black line), MEIC baseline case (blue line), and MEIC updated case (red line) for PM_2.5_, PM_10_, SO_2_, NO_2_, and CO.
