# Supplementary material for: Contributions of Traffic and Industrial Emission Reductions to the Air Quality Improvement after the Lockdown of Wuhan and Neighboring Cities Due to COVID-19
Source: Toxics. 2021 Dec 17;9(12):358. doi: 10.3390/toxics9120358 (PMC8706501; doi:10.3390/toxics9120358)
Supplement: Supplementary file 1 [file toxics-09-00358-s001.zip › toxics-1498494-supplementary.pdf]

toxics

MDPI

Supplementary Materials: Contributions of Traffic and Industrial Emission Reductions to the Air Quality Improvement After the Lockdown of Wuhan and Neighboring Cities due to COVID-19

Xiaoxiao Feng , Xiaole Zhang , Cenlin He and Jing Wang *

1. Satellite Data

The pollutant concentrations can also be observed by satellite, such as National Aeronautics and Space Administration (NASA) Aura Ozone Monitoring Instrument (OMI). The OMI instrument is a UV/VIS solar backscatter spectrometer and can distinguish between gases, such as O_3, NO_2, and SO_2. OMI provides data with a ground resolution of 13 km × 25 km and daily coverage.

To have an overview of air quality before and after the city lockdown, the tropospheric columns of NO_2 observed by OMI from the database of OMI/Aura NO_2 Cloud-Screened Total and Tropospheric Column L3 Global were collected and illustrated within Hubei Province.

Figure S1 shows tropospheric columns of NO_2 on 21, 26, and 28 January. The averaged NO_2 columns in Hubei Province was about 7.16×10^{15} molecule/cm^2 and in Wuhan city about 1.29×10^{16} molecule/cm^2 on 21 January, the day before the lockdown. The averaged NO_2 columns decreased substantially to about 4.00×10^{15}, 3.20×10^{15} molecule/cm^2 in Hubei Province and 4.91×10^{15}, 5.25×10^{15} molecule/cm^2 in Wuhan on 26 and 28 January, respectively, after the lockdown. NO_2 decreased by 49.7% and 60.6% in Hubei Province and Wuhan after the lockdown compared with 21 January. The tropospheric columns of NO_2 reduced significantly after suspending the transportation and shutting down some industry activities.

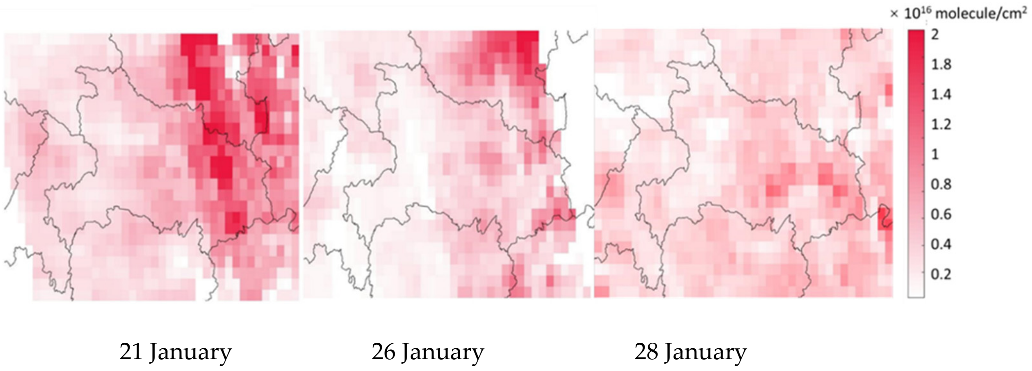

Figure S1. Satellite images. The red profiles refer to tropospheric columns of NO_2 observed by OMI over Hubei Province on 21, 26, and 28 January.

2. Emission Update

The baseline emission inventory MEIC was updated to achieve better model performance. It was assumed that the small change in the emission rate of a single species was linearly proportional to the small change in pollutant concentration. Although the air quality model was non-linear because of the chemical interactions, the linear assumption was widely used in the inverse air pollution modelling of emissions, such as adjusting the emission of NO_x, SO_2, CO, and particulate matter [43–46]. The linear assumption was also applied first and then tested by comparing the observations with the simulation results from the WRF-CMAQ model.

2.1. Method

A linear relation between the change in the emissions and the corresponded change in pollutant concentrations was established, shown in equation (A1).

$$\mathbf{H}\cdot\mathbf{\Delta E}=\mathbf{\Delta C}$$
$$\mathbf{H}=\begin{pmatrix} H_{\text{pm-pm}} & H_{\text{so2-pm}} & H_{\text{nox-pm}} & H_{\text{co-pm}} & H_{\text{voc-pm}} \\ H_{\text{pm-so2}} & H_{\text{so2-so2}} & H_{\text{nox-so2}} & H_{\text{co-so2}} & H_{\text{voc-so2}} \\ H_{\text{pm-nox}} & H_{\text{so2-nox}} & H_{\text{nox-nox}} & H_{\text{co-nox}} & H_{\text{voc-nox}} \\ H_{\text{pm-co}} & H_{\text{so2-co}} & H_{\text{nox-co}} & H_{\text{co-co}} & H_{\text{voc-co}} \\ H_{\text{pm-voc}} & H_{\text{so2-voc}} & H_{\text{nox-voc}} & H_{\text{co-voc}} & H_{\text{voc-voc}} \end{pmatrix} \quad \text{(A1)}$$

In which $H_{a\text{–}b}$ is the entry of the sensitivity matrix H. The subscript a–b denotes the influence of emission a on the concentration of pollutant b. △E is the change in the emission rate. △C is the change in the pollutant concentration.

The sensitivity matrix should be solved at first. Five cases were developed with CMAQ. Each case had different emission rates of pollutant PM, SO_2, NO_x, CO, and VOC with the same domain and meteorological conditions. The change in emission strength △E was known, and the related pollutant concentration △C was calculated by CMAQ. We also had prior knowledge on H. For instance, the emission of particulate matter did not influence the concentration of SO_2. Therefore, $H_{\text{pm–so2}}$ should be 0. Then the sensitivity matrix H was calculated based on the simulation results of the CMAQ cases.

Since the simulation results from CMAQ did not match the observations, the baseline emission inventory should be updated with Equation (A2).

$$\begin{aligned}\mathbf{E}_{update} &= \mathbf{E}_{\text{baseline}}+\mathbf{\Delta E} \\ &= \mathbf{E}_{\text{baseline}}+\mathbf{H}^{-1}\mathbf{\Delta C} \\ &= \mathbf{E}_{\text{baseline}}+\mathbf{H}^{-1}\frac{\mathbf{C}_{\text{baseline}}-\mathbf{C}_{\text{observation}}}{\mathbf{C}_{\text{observation}}}\end{aligned} \quad \text{(A2)}$$

In which E_{update} and E_{baseline} are the updated and baseline emission rate. C_{baseline} is the simulated pollutant concentration with the baseline emission. $C_{\text{observation}}$ is the observed pollutant concentration.

2.2. Results

Based on five simulation results and prior knowledge, the sensitivity matrix was calculated.

$$H=\begin{pmatrix} H_{\text{pm-pm}} & H_{\text{so2-pm}} & H_{\text{nox-pm}} & H_{\text{co-pm}} & H_{\text{voc-pm}} \\ H_{\text{pm-so2}} & H_{\text{so2-so2}} & H_{\text{nox-so2}} & H_{\text{co-so2}} & H_{\text{voc-so2}} \\ H_{\text{pm-nox}} & H_{\text{so2-nox}} & H_{\text{nox-nox}} & H_{\text{co-nox}} & H_{\text{voc-nox}} \\ H_{\text{pm-co}} & H_{\text{so2-co}} & H_{\text{nox-co}} & H_{\text{co-co}} & H_{\text{voc-co}} \\ H_{\text{pm-voc}} & H_{\text{so2-voc}} & H_{\text{nox-voc}} & H_{\text{co-voc}} & H_{\text{voc-voc}} \end{pmatrix}=\begin{pmatrix} 0.33 & 0.28 & 0.10 & 0 & 0.11 \\ 0 & 0.80 & 0 & 0 & 0 \\ 0 & 0 & 0.59 & -0.30 & 1.05 \\ 0 & 0 & 0 & 2.07 & -0.82 \\ 0 & 0 & 0.11 & -0.13 & 1.96 \end{pmatrix} \quad \text{(3A)}$$

The updated emission was calculated. The results show that the baseline emission rate should be multiplied by the factor of 3.5, 0.23, 3.06, and 3.07 for PM, SO_2, NO_x, and CO.

The updated results are shown in Figure S2. Though some periods with high peaks still could not be captured by the simulation, e.g., 15 January for both $PM_{2.5}$ and PM_{10}, the simulation results were improved and had a comparable average as the observations.

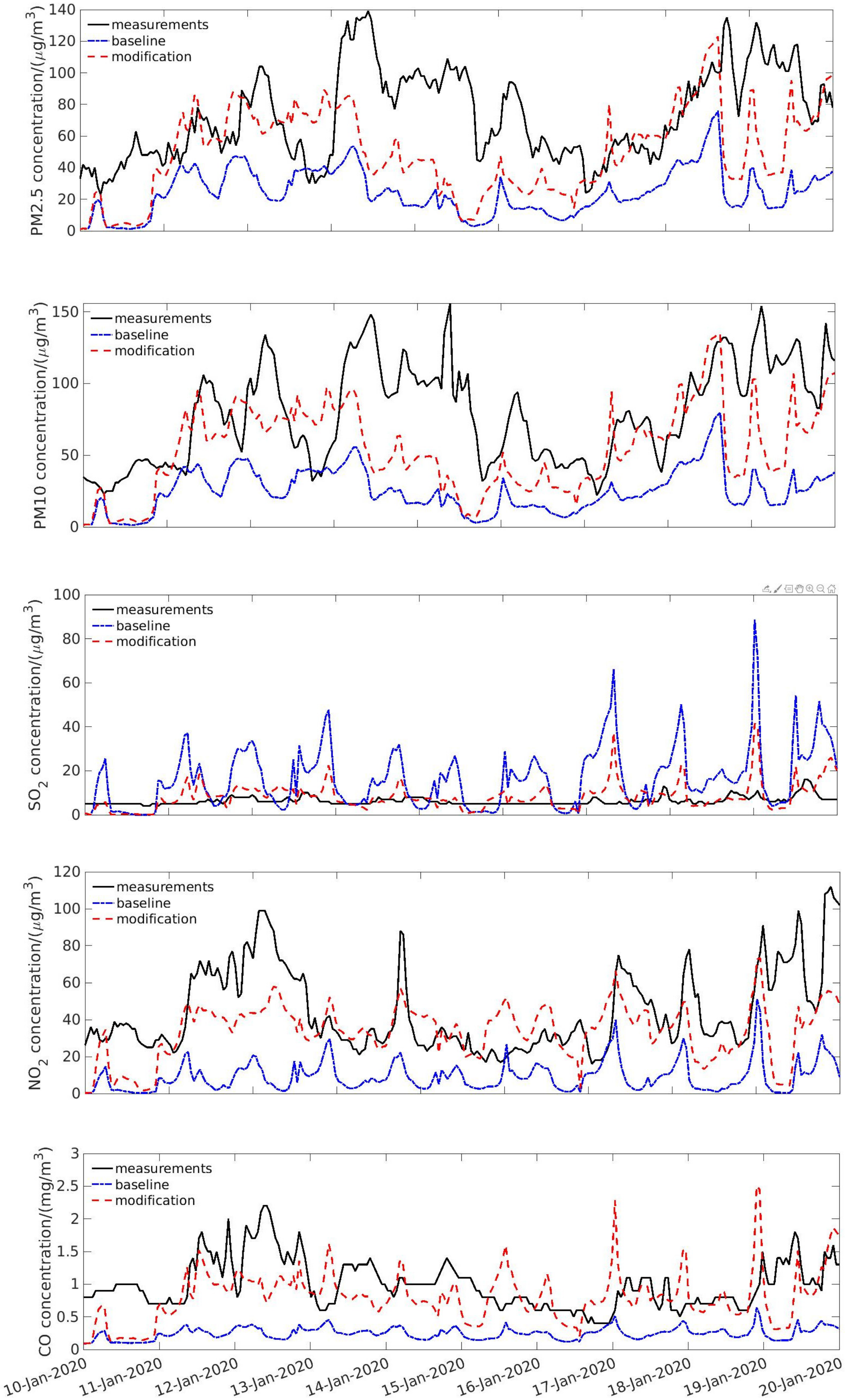

Figure S2. Model validation. Figures above compared the observation (black line), MEIC baseline case (blue line), and MEIC updated case (red line) for $PM_{2.5}$, PM_{10}, SO_2, NO_2, and CO.
